# P-2264. Epidemiology and Outcomes of Bacteremia in Neutropenic Patients: A Changing Scenario over Time

**DOI:** 10.1093/ofid/ofae631.2417

**Published:** 2025-01-29

**Authors:** Fabián Herrera, Diego Torres, Ana Laborde, Lucas Tula, Noelia Mañez, Nadia Suchowiercha, Lorena Berruezo, María L Pereyra, José Benso, Andrea Nenna, María L González Ibáñez, Sandra Lambert, Laura Barcan, Inés Roccia Rossi, Ailén Fernández, Rocío Gago, Verónica Fernández, Vanesa Soto, Natalin Grippo, Magdalena Pennini, Miriam Blanco, Mariángeles Visús, Mariana Reynaldi, Ruth Carbone, Natalia Azula, María Laura Chaves, Fernando Pasteran, Alejandra Corso, Melina Rapoport, Alberto Carena

**Affiliations:** Centro de Educación Médica e Investigaciones Clínicas, CEMIC, BUENOS AIRES, Ciudad Autonoma de Buenos Aires, Argentina; Centro de Educación Médica e Investigaciones Clínicas, CEMIC, BUENOS AIRES, Ciudad Autonoma de Buenos Aires, Argentina; FUNDALEU, Buenos Aires, Ciudad Autonoma de Buenos Aires, Argentina; Hospital El Cruce, Florencio Varela, Buenos Aires, Argentina; Hospital Italiano de Buenos Aires, Sede Central., Buenos Aires, Ciudad Autonoma de Buenos Aires, Argentina; HIGA "Gral. San Martín", La Plata, Buenos Aires, Argentina; HIGA "Dr. Rodolfo Rossi", La Plata, Buenos Aires, Argentina; Hospital Universitario Austral, BUENOS AIRES PILAR, Buenos Aires, Argentina; Hospital Italiano de San Justo, San Justp, Buenos Aires, Argentina; Hospital Municipal de Oncología Marie Curie, Buenos Aires, Ciudad Autonoma de Buenos Aires, Argentina; FUNDALEU, Buenos Aires, Ciudad Autonoma de Buenos Aires, Argentina; Hospital El Cruce, Florencio Varela, Buenos Aires, Argentina; Hospital Italiano de Buenos Aires, Buenos Aires, Ciudad Autonoma de Buenos Aires, Argentina; HIGA "Gral. San Martín", La Plata, Buenos Aires, Argentina; HIGA "Dr. Rodolfo Rossi", La Plata, Buenos Aires, Argentina; Hospital Universitario Austral, BUENOS AIRES PILAR, Buenos Aires, Argentina; Hospital Italiano de Buenos Aires, Sede San Justo, San Justo, Buenos Aires, Argentina; Hospital Municipal de Oncología "Marie Curie", Buenos Aires, Buenos Aires, Argentina; Hospital Universitario CEMIC, Buenos Aires, Ciudad Autonoma de Buenos Aires, Argentina; Stamboulian Laboratories, Buenos Aires, Ciudad Autonoma de Buenos Aires, Argentina; Hospital El Cruce, Florencio Varela, Buenos Aires, Argentina; Hospital Italiano de Buenos Aires, Sede Central., Buenos Aires, Ciudad Autonoma de Buenos Aires, Argentina; HIGA "Gral. San Martín", La Plata, Buenos Aires, Argentina; HIGA "Dr. Rodolfo Rossi", La Plata, Buenos Aires, Argentina; Hospital Universitario Austral, BUENOS AIRES PILAR, Buenos Aires, Argentina; Hospital Municipal de Oncología Marie Curie, Buenos Aires, Ciudad Autonoma de Buenos Aires, Argentina; ANLIS-Malbrán, Buenos Aires, Ciudad Autonoma de Buenos Aires, Argentina; ANLIS-Malbrán, Buenos Aires, Ciudad Autonoma de Buenos Aires, Argentina; ANLIS-Malbrán, Buenos Aires, Ciudad Autonoma de Buenos Aires, Argentina; Hospital Universitario CEMIC, Buenos Aires, Ciudad Autonoma de Buenos Aires, Argentina

## Abstract

**Background:**

The epidemiology of bacteremia (B) in neutropenic patients (NP) may differ according to the geographic region or country and can change over time. The growing rate of multidrug-resistant gram-negative bacilli (MDR-GNB) is usually associated with higher mortality.

**Methods:**

Prospective multicenter study. All the first episodes of B in adult NP with hematologic malignancies were included in 9 centers in Argentina, from May 2014 to December 2023. They were divided in three periods: May 2014-June 2017 (P1), July 2017-June 2020 (P2), and July 2020-December 2023 (P3). Epidemiological, clinical and treatment characteristics, as well as 30-day infection-related mortality of B episodes were compared among the periods.

**Results:**

One thousand three hundred thirty B episodes were included (P1=411, P2=513, and P3=406). Most patients presented high-risk neutropenia (P1=88.3% vs. P2=90.4% vs. P3=90.3%, p=0.50). Septic shock at presentation was P1=21.9% vs. P2=14.8% vs. P3=21.3%, p=0.008. GNB was the leading cause of B, especially in the third period (63.4% vs. 65.5% vs. 79.6%, p< 0.0001). *E. coli*, *Klebsiella* spp., and *P. aeruginosa* were the most prevalent microorganisms, with no differences among the periods. MDR-GNB were more frequent in P1 and P3 compared with P2: 31.6% vs. 31.7% vs. 25.6%, p=0.02. Resistance mechanisms in P1 vs. P2 vs. P3 were as follows: extended-spectrum beta-lactamase production, 26.3% vs. 19.9% vs. 23.5%, p=0.18; KPC production, 7.7% vs. 9.5% vs. 5%, p=0.07; OXA-48 production, 1.5% vs. 0% vs. 1.9%, p=0.05, and MBL production, 0% vs. 0.9% vs. 7.1%, p< 0.0001. Appropriated antibiotic therapy (AT) was higher in P2 and P3 compared with P1: 85.4% vs. 86.1% vs.78.1%, p=0.002. Thirty-day infection-related mortality was P1=16% vs. P2=6.4% vs. P3=9.6%, p< 0.0001.

**Conclusion:**

Both GNB and MDR-GNB were the leading cause of B, with a significant increase in the last three years. A change in carbapenemase type has been observed, with a significant rise in MBL, which presents limited therapeutic options. Notwithstanding that, an improvement in the appropriated AT provided and lower infection-related mortality have been observed in the last few years.

In order to better adapt AT and improve the outcome in NP, changes in B local epidemiology must be known.

**Disclosures:**

Diego Torres, Medical Doctor, Gador: Honoraria|GSK: Advisor/Consultant|Khight Therapeutics: Honoraria|MSD: Honoraria Lucas Tula, Medical Doctor, MSD: Advisor/Consultant|MSD: Honoraria|Takeda Pharmaceuticals: Advisor/Consultant Alberto Carena, n/a, Roche Diagnostics: Latin America Medical Lead for Roche Diagnostics

